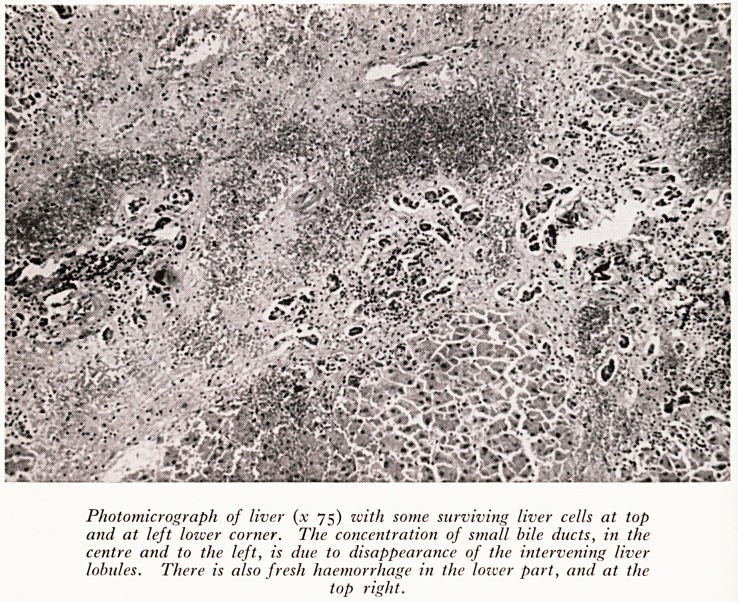# Subacute Hepatitis in a Patient Treated with Parstelin

**Published:** 1963-04

**Authors:** T. F. Hewer


### SUBACUTE HEPATITIS IN A PATIENT TREATED WITH PARSTELIN (P.M. NO. 7209)

*Clinico-Pathological Conference held on 6th March 1962*

CHAIRMAN: PROFESSOR T. F. HEWER

*Dr. Macrae:* This lady, aged 47, was sent to me by Dr. Clarkson as a case of infective hepatitis on 14th October 1961. Her history was that for two or three years she had an enlarged thyroid gland, and increasing nervousness with tremor of her hands for 18 months. She was sent to see Dr. Hemphill in July 1961 and he prescribed Parstelin, 1 tablet twice a day, and she continued to take this drug for the next three months. At the same time he recommended that she should see Dr. Cates since she might have had thyrotoxicosis. Dr. Cates saw her and asked Dr. Tudway to test her radioactive iodine uptake, which turned out to be perfectly normal.

Her more recent history was that for three weeks she complained of upper respiratory infection and ten days before admission developed jaundice with a blotchy, itchy skin rash. She tended to be rather sleepless and was still nervous. She had no abdominal symptoms and no indigestion. On admission her temperature, pulse and respiration were normal; she had an enlarged thyroid with a small hard nodule in it; she was jaundiced; there was a blotchy skin rash which was general; she was quite sensible, well covered, taking her food reasonably well. She was sensible when I talked to her during the day, but became rather rowdy at night and we had considerable difficulty in controlling her. Since she was jaundiced we were not sure what sedation to use, but found that small doses of paraldehyde were sufficient to control her.

The jaundice gradually deepened; she still had no fever; she developed ascites and became rather more difficult to control although she remained quite sensible during the day. Investigations showed that the urine was normal except for a trace of sugar on one occasion. The haemoglobin was 104 per cent; W.B.C. 7,200/cmm (polymorphs 73 per cent). We X-rayed her and found there was no evidence of gall stones or cholecystitis. The serum bilirubin was 17·65 mgm per cent and the alkaline phosphatase 30 units. Serum protein, 6·7 g/100 cc was within normal limits. Thymol turbidity, 9 units, was just a little raised. Serum transaminases of 160 G.O.T. and 280 G.P.T. were both moderately increased. It looked initially like a rather unusual hepatitis. She had ascites; she did not have the early symptoms of infective hepatitis and no dietary troubles at all.

*Professor Hewer:* Did that make you feel that it was perhaps of longer standing than acute hepatitis?

*Dr. Macrae:* I was not very willing to make a diagnosis of straightforward hepatitis and I did wonder what was the significance of this drug Parstelin.

On 25th October we transferred her to the care of Dr. Read who has a special interest in cases of jaundice.

*Dr. Read:* I think first of all I would just like to deal with the drug that Dr. Macrae has mentioned. Just to make things simple, I think it is important to remember that nowadays patients with neurotic and psychotic illnesses are treated with two potentially hepatotoxic groups of new drugs. These are either phenothiazine drugs, of which chlorpromazine is a good example, or hydrazine derivatives, such as Marsilid. The former drugs may cause jaundice in about 1 per cent of cases. They do so by producing intra-hepatic cholestasis; that is by intra-hepatic biliary obstruction. The hydrazine derivatives are mono-amine-oxidase inhibitors; they inhibit the action of enzymes which break down amines to ammonia and aldehydes. They all have this power to a certain degree, but their clinical action is probably not related to it. Marsilid is one of these drugs, which are well known to produce jaundice by liver cell destruction.

This lady had been treated with Parstelin. Parstelin is Parnate (tranylcypromine) plus Stelazine (trifluoperazine) a phenothiazine plus a hydrazine derivative. So here is a lady on a drug of which part is a possible cause of liver cell jaundice, while the other might cause obstructive jaundice. Clinically it is quite obvious that we are dealing with a lady who has liver cell jaundice accompanied by other evidence of hepatocellular failure. She developed ascites and hepatic coma with a positive flocculation test and abnormal plasma proteins.

Dr. Macrae very kindly asked me to see this lady and we transferred her to the B.R.I., realizing, of course, that she was very ill indeed. When we saw her she had the physical findings that he has mentioned. She was very jaundiced, the bilirubin being 25.6 mgm per cent and it is most important to note that 10 mgm of that, which is just under half, was conjugated: the sort of picture that one gets with liver cell jaundice. If there had been much more conjugated bilirubin, just looking at the bilirubin alone, that would be in favour of obstruction. The alkaline phosphatase was 34 (K.A. units), her serum albumin was 2·8 G. Now this illness had been going on for about three weeks, so that the serum albumin would begin to drop because of impaired liver production. Dr. Macrae has mentioned that she was developing fluid retention; in fact she had both ascites and peripheral oedema. Things went from bad to worse; she was very agitated and confused and we had difficulty with the problem of sedation. What is the best sedative to give patients with this sort of condition, realizing that most sedative drugs are possible causes of hepatic coma? We tend to use a long-acting barbiturate because there are potent barbiturate antagonists available should trouble occur. The other thing is, of course, that long-acting barbiturates are mainly excreted through the kidneys and are not broken down in the liver. She did in fact receive phenobarbitone.

The liver size was very important. Clinically the liver was small. That is, by percussion over the right costal margin there was a lessened area of the normal impaired note. In other words, there was clinical evidence of hepatic necrosis and the things that go with hepatic necrosis, namely deep jaundice, fluid retention, the haemorrhagic state and hepatic coma. She had a prothrombin index of 28 per cent, the prothrombin time being 47 seconds and the control on that day 13 seconds, so she was well anticoagulated. Her ascitic fluid contained very little protein—the sort of thing you might get with liver cell failure. She had some signs at the right base, due to a small pleural effusion. The serum proteins, when you looked at them on the strip, showed the reduced albumin, and an alpha 1, alpha 2, beta, and marked gamma increase. Again, this is the sort of pattern that one sees in liver cell necrosis, due to hepatitis or drugs.

The jaundice got worse; she became confused and one morning was found by the houseman in coma. We treated her with the routine sort of therapy that one gives most patients in hepatic coma; that is, we gave her 20 per cent intravenous glucose, neomycin, and large doses of steroids. The reason for the steroids is that they are beneficial in patients with hepatitis. The jaundice decreases and other evidence of liver cell malfunction improves. Here we ran into trouble again, because she had very poor veins, and we had to get the surgeons to cut down to put a polythene catheter up into the superior vena cava. We gave her Vit. $B_6$, pyridoxine. The reason for this is that aminoxidase inhibitors are Vit. $B_6$ inhibitors as well, and although pyridoxine deficiency rarely occurs in man there is good evidence that deficiency can cause neurological sequelae. So we gave it, for what it was worth. We gave her neomycin to sterilize her gut, and, of course, gave her no protein. Dr. Macrae had started these measures before she came to us and we continued, realizing as we did that it was not protein intoxication that was killing her but the fact that she had no, or few, liver cells. Still it is worth treating the "alimentary factor" although one is not treating the major lesion; we could not give her a new liver.

Vit.K was given to try and correct her prothrombin deficiency, or at least to make it less severe. Theoretically it should not be corrected when there is liver cell jaundice, but one is sometimes quite surprised how much the prothrombin time does improve, even in people with liver cell jaundice.

The problem to us, as to Dr. Macrae, was whether this was hepatitis or drug jaundice. In favour of hepatitis there was an epidemic, and there still is, to some degree, an epidemic going on. Against this, she did not have the sort of onset that we like to see, that Dr. Macrae has described; but she did have an illness with 'flu' a week before it began and that could possibly have been the initial illness that one sees in a percentage of patients. Patients who get hepatitis at this age may do rather badly.

Could this lesion have been due to drugs? She was having Parnate and Stelazine, i.e. Parstelin. Parnate has not so far been implicated as a cause of liver cell jaundice, Is there anything in favour of drug jaundice, apart from the fact that she was having the drug? She had this funny rash. Rashes do occur at the beginning of hepatitis sometimes. I would think that an itchy eruption like this, which started before the jaundice and lasted as long as the Parstelin was continued, and cleared up afterwards, is rather in favour of hypersensitivity to a drug. Stelazine can cause a rash and it is possible that the Stelazine did so, while the tranylcypromine caused the liver cell trouble. Throughout this illness there was no pyrexia whatsoever—a fact that has been noted with jaundice due to Marsalid.

She had a leucocytosis of the kind one gets in hepatic necrosis from any cause. So we were left with this difficulty: here was a lady who died with clinical signs of hepatic necrosis of three or four weeks duration. We could not decide whether this was severe hepatitis or drug jaundice due to Parnate, which had caused liver cell damage. She died in hepatic coma. Our diagnosis lay between acute hepatic necrosis due to hepatitis or to damage by a drug of the mono-amine-oxydase inhibitor type, and we hoped that Professor Hewer would agree with the first diagnosis; but we thought he would find it difficult to say which it was, because the histological picture seems to be the same.

*Professor Hewer:* Would Dr. Macrae tell us what he thought about the rash?

*Dr. Macrae:* My first impression of the rash was that it was more like a drug rash than anything else.

*Student:* Was a liver biopsy done?

*Dr. Read:* Oh, I think there is no place whatsoever for a liver biopsy in this case. This is hepatic necrosis. A needle biopsy would have been unnecessary and unwise. She had coagulative trouble; she had a small liver with fluid. These are contra-indications for biopsy. I would have been very annoyed if one of my team had done a liver biopsy on this lady when she was alive; I would have been equally annoyed if they had not attempted one after death, because the histology deteriorates so quickly, and it is important to obtain as good a histological preparation as possible. We tried, after death, but got no liver tissue. This supports the clinical impression that the liver was very small and the needle didn't reach it.

*Professor Hewer:* You do not actually mention the point, but I take it that your fear was that by doing it they might produce a serious haemorrhage?

*Dr. Read:* Oh, yes, and when there is ascites it is very difficult as well as dangerous.

*Professor Hewer:* In any case it won't really help you in your treatment.

*Dr. Read:* No.

*Dr. Draisey:* I have heard it said that, with Ipromazid, jaundice is not due to a primary action of the Ipromazid upon liver cells but that it makes the liver more susceptible to necrosis when the patient gets infectious hepatitis. Do you have any views on this?

*Dr. Read:* That is true. Therefore, we should have had three diagnoses; acute hepatic necrosis due to hepatitis, or to drugs, or to drugs making the way for hepatitis. I agree that some people have written that this can be so. There is a haemagglutination test for hepatitis. It is only positive in about 70 per cent of the cases, but, for what it is worth, cases of drug jaundice show a negative result. Most people are steering away from that view now and think that you have drug jaundice because you are sensitive to drugs, not because it lights up a latent virus or because it makes you more susceptible to it. But it is, as you say, a third possible theory.

*Dr. McConnell:* Is it ever possible to isolate the virus of infectious hepatitis from a patient in life?

*Professor Hewer:* It has never been grown, I believe, but it can be transmitted to volunteers who ingest a cell-free filtrate.

*Dr. McConnell:* Yes, I know that. But it has never been cultured artificially?

*Dr. Macrae:* As far as I know it has not.

*Dr. Read:* I thought it had, sir. I think American workers claim to have isolated it recently by tissue culture and to have transmitted it to humans.

*Professor Hewer:* The Detroit workers have not yet, I believe, published their details and it has not been confirmed.

*Dr. Lloyd:* Has Dr. Read any objection to paraldehyde as a sedative, apart from the fact that you cannot "turn it off"?

*Dr. Read:* Not apart from that.

*Dr. Lloyd:* I am rather surprised that Vitamin K therapy should be effective, when there is severe liver cell damage. Vitamin K gets converted into prothrombin by the liver cells; yet, in spite of the fact that there is in this condition hardly any functioning liver tissue, you find that administration of Vitamin K shortens the prothrombin time. This must mean that some conversion to prothrombin is taking place.

*Dr. Read:* I cannot explain it but I believe it happens. When we have someone with hepatitis or cirrhosis, and we want a liver biopsy, but the prothrombin time is low, we find that Vitamin K raises it to a normal figure. Somehow it works: I do not know why.

*Dr. Lloyd:* Is it possible that the synthesis is done somewhere other than in the liver?

*Dr. Read:* I don't know.

*Professor Hewer:* It is not entirely surprising because you would need to have an almost complete destruction of all the liver cells for no prothrombin synthesis to take place in that organ. I do not know how many survivors are necessary but I shall show you in a moment that there were still some liver cells alive. It could have been worse!

*Dr. Read:* Well, not much, sir.

*Dr. Lewis:* I think that is the accepted explanation, because you have to lower the prothrombin a lot to affect the one-stage prothrombin clotting time and it only needs a small increase of prothrombin to restore it to the normal range. If you have any functioning liver cells they may be able to synthesise just sufficient to bring the prothrombin time within this range after giving Vitamin K. I do not think it is necessary to postulate synthesis of prothrombin elsewhere.

*Student:* Dr. Read, are certain people sensitive to this sort of drug, or are there some who can go on taking it for a long period?

### PLATE XV

*Posterior surface of liver showing small projecting nodules of surviving and regenerating liver cells on a flat background of largely necrotic liver.*

### PLATE XVI

*Cut surface of liver showing pale surviving nodules of liver cells and dark haemorrhagic areas.*

PLATE XVII

*Photomicrograph of liver (x 75) with some surviving liver cells at top and at left lower corner. The concentration of small bile ducts, in the centre and to the left, is due to disappearance of the intervening liver lobules. There is also fresh haemorrhage in the lower part, and at the top right.*

*Dr. Read:* Hypersensitivity is important in drug induced jaundice. Only about 1 per cent of patients get chlorpromazine jaundice, and only about 1 per cent of patients treated with Marsilid get Marsilid jaundice. There is a hypersensitivity in these patients. Of course, the fact that this lady had a drug rash may suggest that she was that type of person.

*Dr. Lewis:* Has anybody tried to test for an antigen-antibody reaction in the serum of these cases in the same way as Ackroyd showed in rubella and also in the Sedormid purpuras, where there is an antibody developed in the serum against a complex of platelets and the offending drug?

*Dr. Read:* No, I do not think they have. In hepatitis of course there is good evidence that there are complement fixing antibodies against liver cells and other tissue in the serum, so that autoimmune damage could occur, but I don't know of any work suggesting antibody formation against hepatotoxic drugs.

*Dr. Sanerkin:* These drugs are all in common use, are they not, Dr. Read?

*Dr. Read:* Oh, in very common use. Dr. Hemphill has been using Parnate at Barrow on a large series of cases and I think in fairness to Parnate and to Dr. Hemphill, he has not run into any sort of trouble.

*Student:* Dr. Read, in cases of jaundice due to monoaminoxidase inhibitors what is the fatality rate?

*Dr. Read:* If a patient develops jaundice from one of these drugs there is far more chance of a fatal outcome than if there is hepatitis. The mortality rate in this sort of thing can be as high as 20 per cent from monoaminoxidase inhibiors

*Professor Hewer:* I think I should now show you the post-mortem findings. She was obese and had a branny desquamation of the skin. There was cyanosis, deep jaundice, and generalized oedema, with ascites and a little fluid in each pleural cavity. The liver (Plates XV and XVI) was extremely shrunken and soft, with a wrinkled surface. It weighed only 840 grams, less than half the normal, and on section was of an almost fluid consistency, with a few small nodules of apparently living and regenerating liver just beneath the capsule, and in a few places in the centre. Otherwise the cut surface showed fresh haemorrhages and no sign of bile duct obstruction. The gall-bladder was full of dark viscid bile, with no calculi.

Microscopically, blocks from three different parts of the liver showed a uniform picture of an early cirrhosis with a few areas of nodular regeneration against a background of recent haemorrhagic necrosis which is clearly centrilobular in distribution (Plate XVII). The cirrhotic scarring is slight and could well be of no more than six or eight weeks duration, while the necrosis is quite recent. There is no cellular infiltration. A few bile canaliculi in the surviving areas of liver contain plugs of bile. The absence of cellular infiltration might be due to the steroid therapy that she received. The spleen was acutely congested as a result of thrombosis of the splenic vein. The portal vein and its other tributaries were patent. The stomach showed many small acute ulcers. The thyroid was moderately and uniformly enlarged and microscopically had very much the appearance of a primary toxic goitre, with considerable lymphocytic infiltration and acini with little or no colloid and high cubical epithelium.

I concluded that this was best described as a subacute necrosis of the liver which began as a centrilobular necrosis. It could have been due to infective hepatitis or to a toxic drug but I think the former cause more likely. I am rather uncertain about the thyroid since we are told that there was no clinical evidence of hyperthyroidism, and yet the histological appearance does suggest it. Hyperthyroidism does make the liver more vulnerable.

*Dr. Read:* Another diagnosis we must consider is homologous serum hepatitis. This disease is more likely to have an insidious onset, as it did with this patient, and to be more severe than the ordinary infective hepatitis. This was a "hospital illness"

and one has to remember the possibility. Do you think the centrilobular necrosis indicates a hepatitis rather than a drug necrosis?

*Professor Hewer:* I don't think so. You get centrilobular necrosis in so many things. Many drugs do it, and whether it is due to a virus or to a drug the liver cells curl up and die and there is some degree of cellular infiltration in either case. There is cellular infiltration, for instance, in carbon tetrachloride poisoning if it is not immediately lethal.

*Dr. Read.* I was rather meaning this group of drugs, the monoamine oxidase inhibitors, as against hepatitis.

*Professor Hewer:* I have not seen enough of them but my impression from the literature is that it is just about the same.

*Dr. Lloyd.* Do any of these drugs block the formation of thyroxine, and could that be the explanation of the changes in the thyroid?

*Dr. Read:* I do not know off-hand, but I do not think so. They do of course block the action of Vitamin $B_6$. One interesting point is that Marsilid has been suggested as a treatment for hepatic coma, the theory here being that Marsilid, as an aminoxidase inhibitor, inhibits the breakdown of amines to ammonia and aldehyde; and ammonia is, of course, one of the substances which has been thought to be toxic. It is not the only one; it is a convenient one to measure in the blood. Marsilid does not improve patients with hepatic coma although it does lower the blood ammonia. The precursors of ammonia are probably as much to do with hepatic coma as ammonia itself, so perhaps this is not unexpected.

*Student:* What is the mortality from chlorpromazine jaundice?

*Dr. Read:* There should not be a mortality—provided you can keep the surgeons away! I personally have seen chlorpromazine jaundice going on for two years. These patients, of course, get a lot of trouble, loss of weight, steatorrhoea, Vitamin K deficiency, low serum proteins, tremendous itching, etc., but they should make a perfect recovery and there is no evidence that biliary cirrhosis follows this sort of picture. There are fatal cases recorded but these are nearly always debilitated patients, particularly in mental hospitals, and usually with some complicating physical illness. They may well die, but this mortality otherwise from chlorpromazine jaundice should not be high.

*Dr. Lloyd:* I suppose they never die from hepatic coma?

*Dr. Read:* No, they do not.

*Professor Hewer:* We conclude then that this woman developed an acute hepatitis, which became subacute, at a time when there was an epidemic of infective hepatitis, but she had been receiving a drug which is suspected of a capacity to produce necrosis of the liver cells, and we do not really know how to apportion the blame.

## Figures and Tables

**Figure f1:**
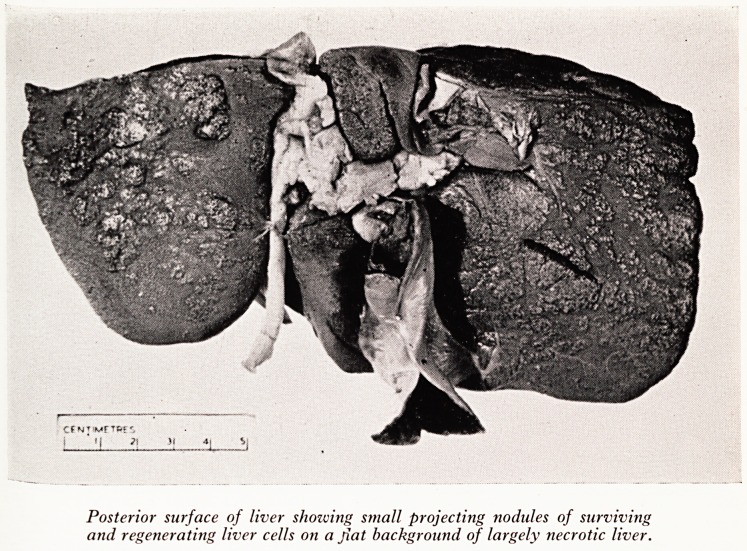


**Figure f2:**
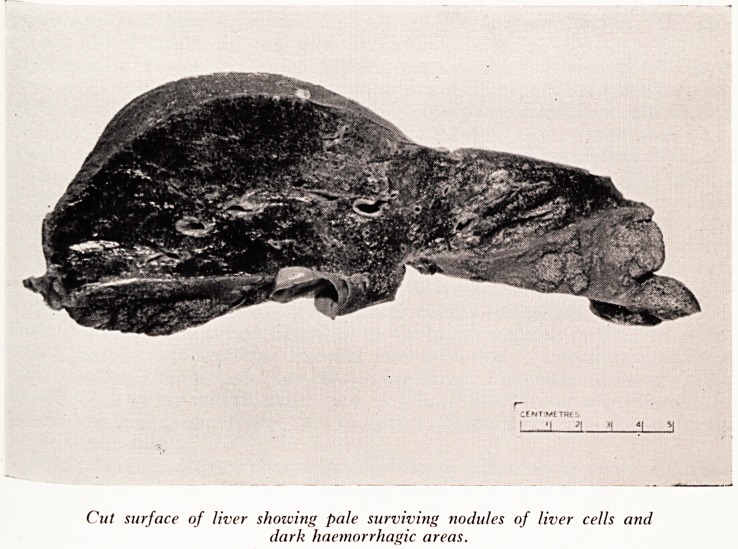


**Figure f3:**